# Supplementary material for: Trends and outcomes of percutaneous coronary intervention during the COVID-19 pandemic in Michigan
Source: PLoS One. 2022 Sep 26;17(9):e0273638. doi: 10.1371/journal.pone.0273638 (PMC9512204; doi:10.1371/journal.pone.0273638)
Supplement: Attachment — Submitted filename: Response to reviewersPLOShg.docx [file pone.0273638.s005.docx]

**Reviewer #1: Azzalini et al. report an interesting study regarding the evaluation of the influence of the COVID-19 pandemic on temporal trends and outcomes of patients undergoing percutaneous coronary intervention (PCI) during the year 2020 compared to year before (2019). The topic is of high interest due the persistence of the COVID pandemic that is bringing the world health to its knees. Despite numerous previous manuscripts depicting differences in PCI volumes and outcomes between the pandemic and the pre-pandemic era this paper covers a longer period compared to initial reports that exclusively focused on the “first wave” of the pandemic. Furthermore, it gives in-depth insights on the outcomes of all-comers undergoing PCI before and during the COVID-19 pandemic, showing that the worse in-hospital outcomes are not exclusively related to the COVID infection itself but also probably to the “collateral damage” of the COVID-19 pandemic to the entire cardiac care system.
The manuscript is well written, very interesting with a robust statistical methodology. Therefore, I do not have further revisions to propose.**

Thank you for your kind commentary.

**Reviewer #2: The aim of this retrospective, multicenter study was to assess outcomes of patients undergoing percutaneous coronary intervention (PCI) in Michigan during the COVID-19 pandemic in 2020 (between March and December), comparing them to outcomes in the same period of the previous year. The Authors used data from the Blue Cross Blue Shield of Michigan Cardiovascular Consortium (BMC2) PCI registry which included data from all the catheterization laboratories in Michigan. The Authors found a ~15% decrease in overall PCI volume from the pre-pandemic to the pandemic period; monthly mortality rates for primary PCI were in general higher in the pandemic period. Furthermore, patients undergoing PCI in the pre-pandemic period, those undergoing PCI in the pandemic period had a higher risk of death. This last finding was more pronounced in patients presenting with ST-segment elevation myocardial infarction (STEMI).
The topic is interesting, the analyses are elegant and the manuscript clearly written.**

**I have some considerations:
• Monthly mortality rates for primary PCI were in general higher in the pandemic period, a finding that was however not related to clinically significant system delays in STEMI care. As already mentioned by the Authors, one of the possible interpretations is that patients tried to avoid contacting the emergency services for fear of entering the hospitals. Indeed, it is possible that predominantly patients with very severe symptomatology contacted the emergency services or went to the hospital. In this context, it is also possible that patients waited until they had an advanced clinical situation before asking for assistance. It is known that several patients who were not infected with COVID-19 suffered complications of myocardial infarction associated with late presentation. It would be interesting to add “pain to balloon” to the data shown.**

We agree with the reviewer that this is likely possibility. However we are not able ascertain this since the BMC2 database did not capture pain onset time and hence is unable to calculate the pain to balloon time.

**• It is important to note also the low absolute number of STEMIs and NSTEMIs in 2020 compared to 2019: perhaps another indication that many patients stayed home and only the most symptomatic called for rescue.**

This is indeed an important consideration, and it has been underlined in the discussion (page 18 and page 19). As the reviewer suggests, the lower number of ACS in 2020 might have been driven by the fact that patients tended to stay home for fear of contracting COVID-19 in the hospital: these considerations are discussed at the end of page 19.

**• The authors performed outcome analyses using in-hospital mortality. Although the latter is widely used in the literature, I believe it would be more elegant and objective to use 30-day mortality, if it were available.**

We agree with the reviewer that 30 day mortality can provide additional information compared with in hospital mortality. Unfortunately, the BMC2 registry collects only in-hospital data and all reported outcomes accordingly reflect in-hospital events.
